# Clinical Utility of Neutrophil CD64 to Detect Extrapulmonary Tuberculosis in Three Patients with Rheumatoid Arthritis Undergoing Treatment with Biologics

**DOI:** 10.1155/2018/2856546

**Published:** 2018-12-06

**Authors:** Shinichi Nogi, Yoshiyuki Arinuma, Akiko Komiya, Atsushi Hashimoto, Toshihiro Matsui, Shigeto Tohma

**Affiliations:** ^1^Department of Rheumatology, National Hospital Organization Sagamihara National Hospital, Sagamihara, Kanagawa, Japan; ^2^Department of Rheumatology and Infectious Diseases, Kitasato University School of Medicine, Sagamihara, Kanagawa, Japan; ^3^Department of Clinical Laboratory, National Hospital Organization Sagamihara National Hospital, Sagamihara, Kanagawa, Japan; ^4^President, National Hospital Organization Tokyo National Hospital, Tokyo, Japan

## Abstract

Biologics play a key role in the treatment of rheumatoid arthritis (RA), while RA-related serious infection remains an unsettled clinical problem. Detection of tuberculosis (TB) is challenging due to the difficulty in distinguishing symptoms such as fever and elevation of inflammatory markers from other infections or a disease flare of RA. The expression of the CD64 molecule on neutrophils (neutrophil CD64) was upregulated by various infections including TB. However, it was not affected by disease activity of RA or by any therapy against RA. The present article reports three cases of extrapulmonary TB which occurred in patients with RA undergoing treatment with biologics. The marked increase in the levels of neutrophil CD64 may provide important insight into the diagnosis of TB.

## 1. Introduction

The use of biologics resulted in a paradigm shift in the treatment of rheumatoid arthritis (RA). However, concern regarding the increase in the incidence of serious infection remains [1, 2]. In particular, the reactivation of latent tuberculosis infection (LTBI) is a clinical challenge due to its atypical clinical features (miliary or extrapulmonary presentations) [3]. The risks of developing extrapulmonary tuberculosis were significantly higher in RA patients [4]. Early detection of tuberculosis (TB) is challenging due to the difficulty in distinguishing symptoms such as fever and elevation of inflammatory markers from other infections or a disease flare of RA.

The property of CD64 molecule on neutrophils (neutrophil CD64) has been utilized as a diagnostic marker of infection particularly in sepsis [5]. Previous studies have reported that expression of the neutrophil CD64 was upregulated by various infectious diseases including TB and was not affected by RA disease activity, nor the use of corticosteroids, disease modifying antirheumatic drugs (DMARDs), or biologics including tocilizumab [6, 7]. In addition, the levels of neutrophil CD64 in TB were markedly higher than those observed in other infections [6]. Neutrophil CD64 as well as TLR2 and TLR4 was increased in pulmonary tuberculosis patients compared to healthy volunteers [8].

The present article reports three cases of extrapulmonary TB which occurred in patients with RA undergoing treatment with biologics. The marked increase in the levels of neutrophil CD64 may provide important insights into the diagnosis of TB.

## 2. Case Presentation

### 2.1. Case 1

**Figure 1:**
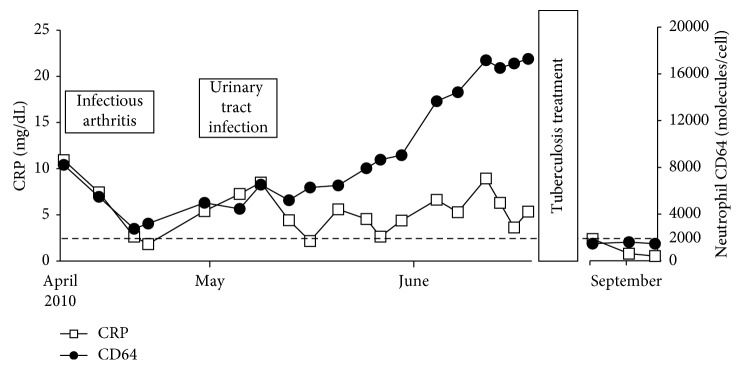
Levels of neutrophil CD64 elevated with dissociation of a CRP change (case 1). The cutoff value for the levels of neutrophil CD64 was 2000 molecules/cell.

**Figure 2:**
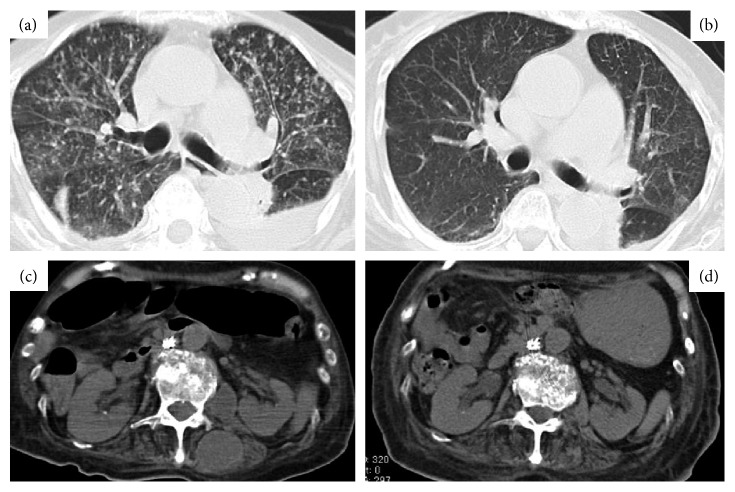
Computed tomography scan images of case 1. Particulate lesions diffusely observed in both lung fields (a) were diminished after antituberculosis therapy (b); cystic lesion with the same absorption range as abscess observed in paraspinal muscle (c) was also diminished after antituberculosis therapy (d).

A 74-year-old female was suffering from RA since 1993. The patient had received treatment with various DMARDs such as methotrexate (MTX), salazosulfapyridine (SASP), gold sodium thiomalate, and bucillamine (BUC). However, the administration of these agents was discontinued due to treatment-related adverse events or insufficient efficacy. In March 2009, adalimumab (ADA) (40 mg every 2 weeks) was added to low-dose prednisolone (PSL). The patient initiated prophylactic therapy with isonicotinic acid hydrazide (INH) for 6 months targeting recurrence of old pulmonary TB. A tuberculin skin test (TST) (positive) was performed prior to initiation of treatment with ADA. However, in August 2009, ADA was switched to etanercept (ETN) due to secondary failure to ADA. In October 2009, the patient was admitted to the National Sagamihara Hospital with infectious arthritis of knee or ankle caused by methicillin-resistant *Staphylococcus aureus* (MRSA). In April 2010, intravenous administration of vancomycin or teicoplanin (TEIC) improved the aforementioned symptoms (Figure 1). RA disease activity was low with PSL 10 mg/day and SASP 1 g/day without ETN. In May 2010, the patient presented with urinary tract infection (UTI) caused by *Escherichia coli* with an observed increase in the levels of neutrophil CD64. Neutrophil surface CD64 expression was measured using QuantiBRITE CD64 PE/CD45 PerCP (Becton Dickinson, San Jose, CA) and a FACSCalibur flow cytometer (Becton Dickinson) as previously described [6]. The UTI improved following appropriate treatment for pathogenic bacteria. However, the levels of neutrophil CD64 continued to increase reaching >10,000 molecules/cell in June 2010. Subsequently, the patient had fever with bilateral infiltrative shadows appearing on a chest X-ray and positive MRSA sputum culture. TEIC was administered intravenously and the patient's temperature returned to normal, with a decrease in the levels of serum C-reactive protein (CRP). However, the levels of neutrophil CD64 increased up to 17,509 molecules/cell, and newly developed diffuse reticulonodular shadows were observed in both lung fields using a chest X-ray. Whole-body computed tomography (CT) revealed diffuse small granular shadows on both lungs and fluid accumulations (abscess) around the lumbar vertebrae Figures 2 and 2. TST was negative, and the smears of sputum and gastric juice were negative for acid-fast bacilli. However, the smear of specimens obtained from the abscess by echo-guided percutaneous drainage contained acid-fast bacilli identified as *Mycobacterium tuberculosis* by polymerase chain reaction (PCR). The patient was diagnosed with miliary TB along with tuberculous spondylitis and transferred to a hospital which had a TB ward. In September 2010, after treatment for TB, the levels of neutrophil CD64 decreased below the cutoff value (<2000 molecules/cell) and abnormal findings on the CT scan disappeared Figures 2 and 2. Sustained high levels of neutrophil CD64 were considered to be due to reactivation of LTBI.

### 2.2. Case 2

**Figure 3:**
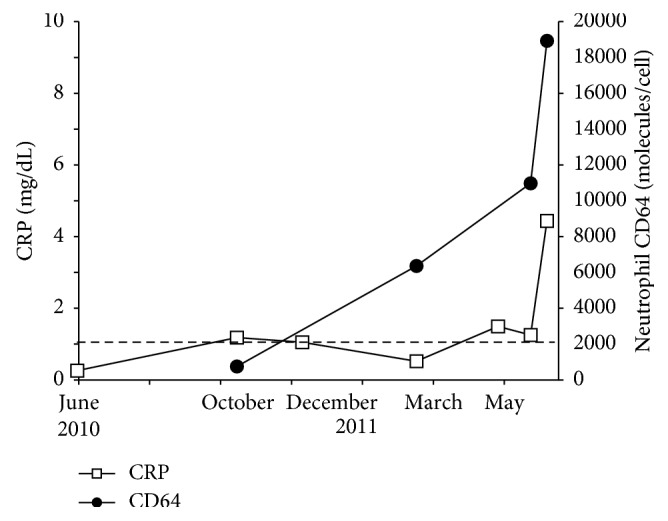
Levels of neutrophil CD64 increased even after antibiotic therapy (case 2).

**Figure 4:**
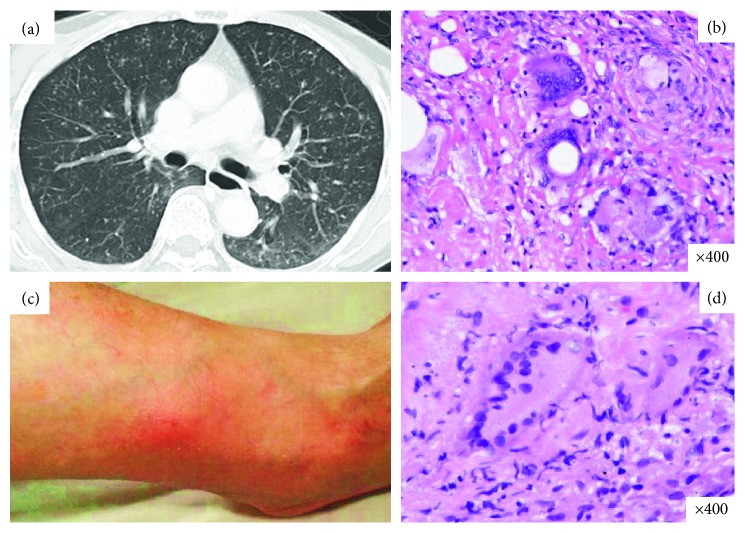
(a) Chest CT scan image at the time of admission. Diffuse particulate shadows are shown in the whole-lung field at random; (b) noncaseating epithelioid granuloma was observed in bronchial and alveolar specimens obtained through bronchoscopy (hematoxylin and eosin (H & E) stain; magnification x400); (c) a cellulitis-like lesion on the patient's right lower limb; (d) biopsy specimens from cellulitis also revealed erythema induratum of Bazin (H & E stain; magnification x400) (case 2).

A 73-year-old female was suffering from RA since 1983. In December 2008, administration of ADA 40 mg every 2 weeks was initiated due to insufficient efficacy of conventional DMARDs. Prior to initiation of ADA, a positive TST was obtained, and the patient received INH for 9 months. ADA therapy was effective against RA. However, in February 2010, she returned to the hospital with cellulitis on both legs. The cellulitis improved with administration of antibiotics (imipenem) and temporary discontinuation of ADA. In May 2011, treatment with antibiotics was associated with slight fever, elevated serum CRP, and continuous elevation of neutrophil CD64 (up to 18,831 molecules/cell) (Figure 3). ADA was discontinued and further examination revealed diffuse reticulonodular shadows in the lung fields through chest X-ray and CT scan, suggesting the development of miliary TB (Figure 4). Acid-fast bacilli were observed in smear preparations from sputum and gastric juice, and a sputum PCR test was positive for *Mycobacterium tuberculosis*. The patient was transferred to a hospital which had a TB ward. Noncaseating epithelioid granuloma was observed in bronchial and alveolar specimens obtained through bronchoscopy (Figure 4). Biopsy specimens from a cellulitis-like lesion on the patient's right lower limb (Figure 4) also revealed erythema induratum of Bazin (Figure 4). The levels of neutrophil CD64 decreased below the cutoff value after anti-TB treatment with improvement of clinical manifestations.

### 2.3. Case 3

**Figure 5:**
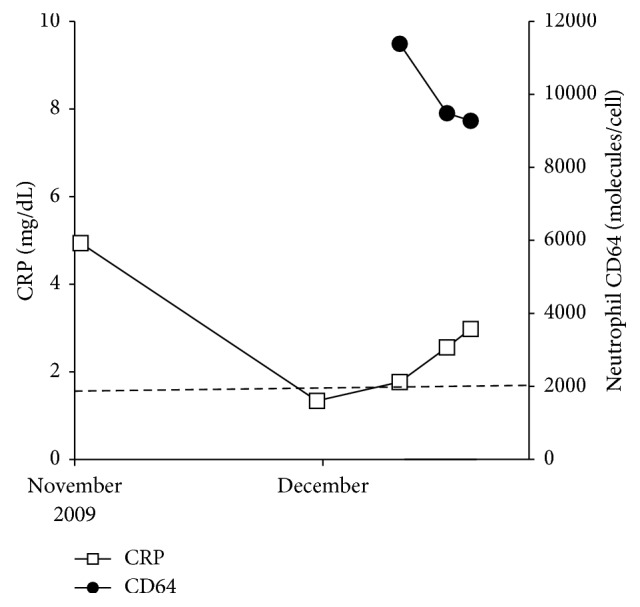
High levels of neutrophil CD64 were maintained since admission to the hospital (case 3).

**Figure 6:**
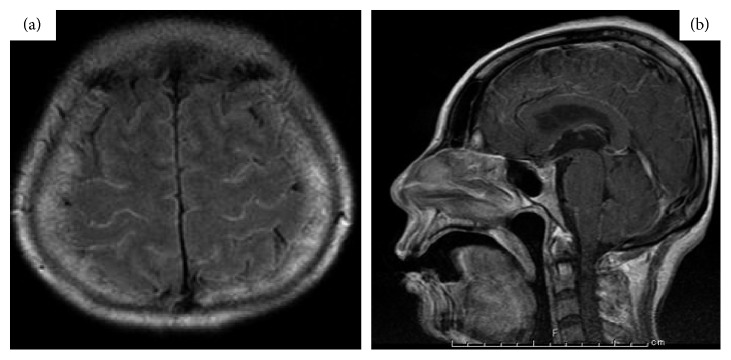
(a, b) Fluid-attenuated inversion recovery (FLAIR) magnetic resonance imaging of the brain revealed pia-subarachnoid enhancement (case 3).

A 49-year-old female was suffering from RA since 2004. Initially, the patient received SASP and BUC. However, therapy was switched to MTX in 2007 due to insufficient efficacy. In 2009, ETN was administered without chest X-ray and TST examinations by a previous clinic doctor and was effective against RA. In October 2009, the patient visited National Sagamihara hospital and had fever (>38°C), headache, and general malaise. Although ETN was discontinued and PSL (10 mg/day) was administered, the fever persisted, the headache worsened, and vomiting was reported. The patient was admitted to the National Sagamihara Hospital in December 2009. Body temperature was approximately 40°C without manifestations on physical examinations. The levels of serum CRP were approximately 1 mg/dl, and the levels of neutrophil CD64 were highly elevated (up to 11,387 molecules/cell) (Figure 5). TST was negative, and head CT scan images showed no abnormalities. A lumbar puncture revealed slightly turbid cerebrospinal fluid (CSF) with 344 white blood cells/*μ*l (88% lymphocytes and 12% polymorphs) and low levels of glucose (29.9 mg/dl in CSF vs. 116 mg/dl) in blood. Assessment for cryptococcal antigen in the CSF was negative. Acid-fast bacteria were not detected in smear preparations from CSF and gastric juice. The following day after admission, the patient's conscious level worsened. Gd-DTPA-enhanced magnetic resonance imaging (MRI) of the brain was performed. A pia-subarachnoid enhancement was found on the fluid-attenuated inversion recovery (FLAIR) sequence, which was compatible with meningitis (Figure 6). Initially, acyclovir was administered; however, the fever and level of consciousness were not improved. A lumbar puncture was performed again four days later and acid-fast bacteria were not found in the smear preparation. PCR of CSF for *Mycobacterium tuberculosis* was positive. The patient was diagnosed with tuberculous meningitis and transferred to a university hospital. Combination therapy of INH, rifampicin, and ethambutol resulted in gradual improvement of the level of consciousness. Six weeks later, positive results for *Mycobacterium tuberculosis* of cultures of gastric juice and CSF were reported. The levels of neutrophil CD64 decreased below the cutoff value after completion of anti-TB therapy.

## 3. Discussion

The present article reports three cases with RA in whom high expression of neutrophil CD64 may provide important insight into the diagnosis of extrapulmonary TB occurring during treatment with biologics. Cases 1 and 2 received INH as prophylactic therapy for TB, while cases 1 and 3 showed negative TST results at the time of TB diagnosis. All cases did not show typical findings of TB and the diagnoses were challenging.

TB is a common infectious disease in Japan. In 2014, the incidence of TB was 18 per 100,000 individuals in Japan, five-fold higher than in the United States, and three-fold higher than in Canada or Europe [9]. TB is regarded as a “reemerging infectious disease” in the developed world, and countries including Japan have proposed guidelines for the management of LTBI. Although it is recommended that TST or interferon-γ release assay (IGRA) is performed in patients prior to the use of biologics [10], false-negative results in TST or IGRA may occasionally be observed in immunosuppressed patients treated with biologics, corticosteroids, or DMARDs. Moreover, it is well known that the results of TST may be false-negative in cases of miliary TB (case 1). Furthermore, atypical clinical features of active TB (miliary or extrapulmonary presentations) are common in patients undergoing treatment with biologics [3]. Screening tests prior to the administration of biologics may reduce the risk of reactivation of LTBI [11]. Park et al. reported that serial TST combined with IGRA may be useful for the identification of false-negative results for LTBI and new TB infections in patients with immune-mediated inflammatory diseases undergoing long-term anti-TNF therapy [12]. In addition, studies reported that the upregulation of neutrophil CD64 may be helpful for the detection of infectious diseases including TB and may distinguish infection from a flare of RA even in patients treated with biologics [6, 7]. In patients with RA, the sensitivity and specificity of the levels of neutrophil CD64 for the diagnosis of infection (using a cutoff value of 2000 molecules/cell) were 92.7% and 96.5%, respectively. Additionally, the expression levels of neutrophil CD64 in TB tended to be much higher than those observed in other infections [6]. Cases 1 and 2 were very complicated due to another underlying remitting bacterial infection which elevated the levels of neutrophil CD64 prior to TB activation. The levels of neutrophil CD64 remained high (>10,000 molecules/cell) despite appropriate antibiotic treatment, indicating reactivation of LTBI. Although the levels of CRP fluctuated by various bacterial infections in the present cases, the levels of neutrophil CD64 remained high. These findings reflected the activity of underlying TB and distinguished TB from other bacterial infections.

In cases suspected of LTBI, prophylactic therapy against TB (mainly using INH) is recommended for 6 to 9 months. This therapy reduces the incidence of TB in patients with RA treated with biologics [11]. However, extended use of INH (>9 months) may prevent reactivation of LTBI. Cases 1 and 2 developed TB as reactivation of LTBI despite the recommended precautionary regimen using INH. It was reported that out of 36 LTBI patients with rheumatic diseases receiving anti-TNF drug therapy, seven patients developed active TB despite receiving or completing the anti-TB chemoprophylaxis regimen [13]. Treating physicians ought to consider reactivation of LTBI in patients undergoing treatment with biologics. For this reason, examinations for TB (chest X-ray and TST or IGRA test) must be performed before starting biological therapy. Additionally, determining the levels of neutrophil CD64 may be a useful and supportive examination for the early detection of TB.

In the three cases presented herein, treated with biologics such as ETN and ADA, neutrophil CD64 was useful for the detection of TB. Tocilizumab (TCZ), an antiinterleukin-6 receptor antibody, suppresses inflammatory responses such as fever and elevation of CRP levels. Hence, this infection may be overlooked in patients treated with TCZ. Previous studies reported that the levels of neutrophil CD64 may be increased by various infections in patients with RA undergoing treatment with TCZ, although the fever and elevation of CRP levels were completely masked [7]. Furthermore, the usefulness of neutrophil CD64 as an infection marker in patients undergoing treatment with other biologics (golimumab, abatacept, and certolizumab pegol) has been demonstrated (data not shown). Infection, including TB, is the most pivotal adverse event in patients with RA receiving treatment with biologics. Moreover, the levels of neutrophil CD64 are not affected by RA disease activity. Therefore, serial monitoring of the levels of neutrophil CD64 may be useful for the early detection of infection including TB in patients with RA undergoing treatment with biologics.

The levels of neutrophil CD64 may not be highly increased by only tuberculosis, but also conditions other than infection, such as adult-onset Still's disease [14], interstitial pneumonia [6], vasculitis [6, 15], and malignant lymphoma [16]. Accordingly, it is very important to exclude these differential diagnoses of TB in patients with elevated levels of neutrophil CD64. Additionally, these findings support the necessity of serial monitoring of neutrophil CD64 levels.

In conclusion, the cases presented herein emphasize the usefulness of serial monitoring of the levels of neutrophil CD64 for the detection of TB in patients with RA undergoing treatment with biologics.
